# Supplementary material for: CDK4/6-dependent activation of DUB3 regulates cancer metastasis through SNAIL1
Source: Nat Commun. 2017 Jan 9;8:13923. doi: 10.1038/ncomms13923 (PMC5228031; doi:10.1038/ncomms13923)
Supplement: Supplementary Information — Supplementary Figures 1-11, Supplementary Methods [file ncomms13923-s1.pdf]

Supplementary Fig. 1

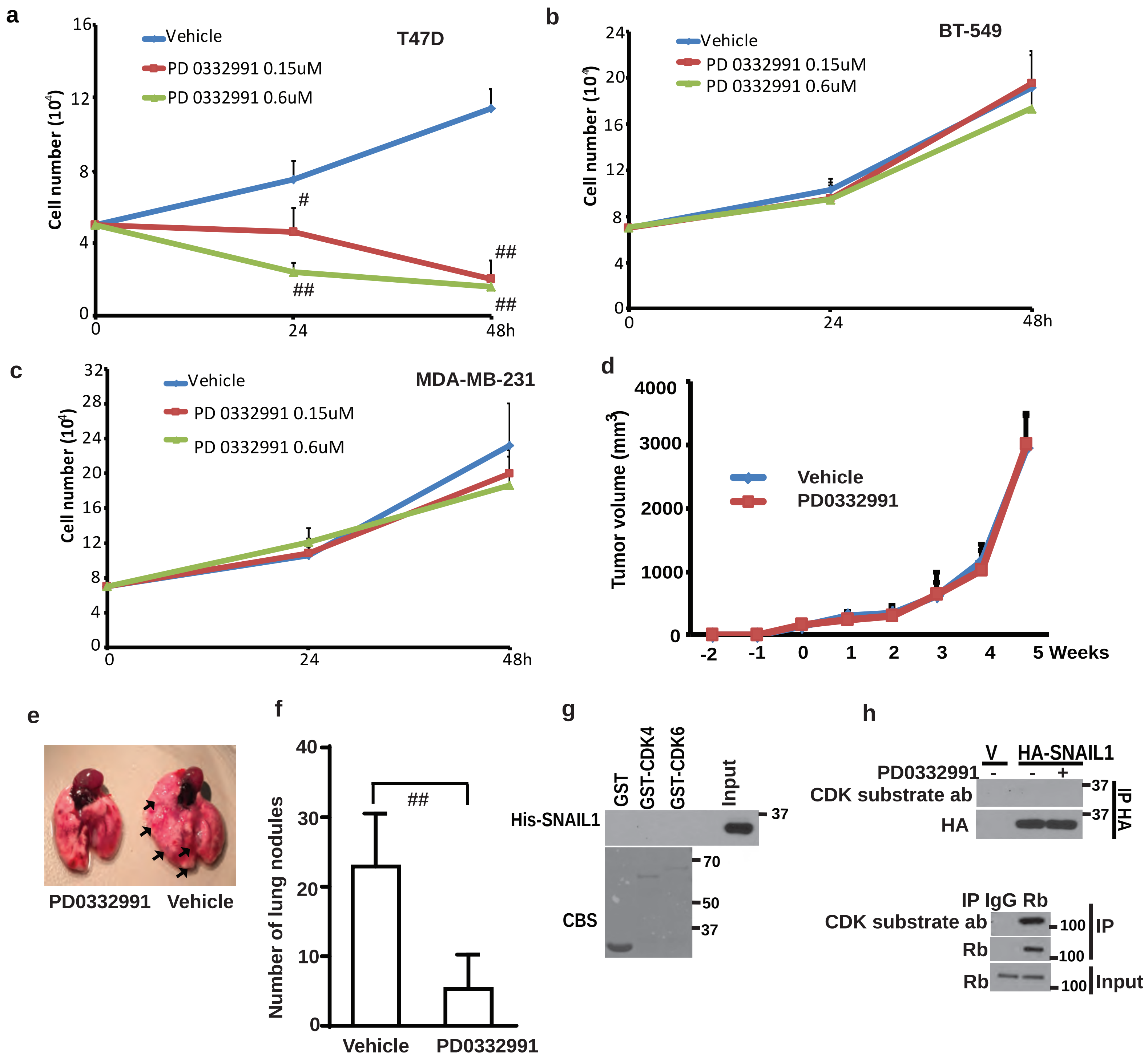

Supplementary Figure 1. CDK4/6 inhibitor PD0332991 does not affect the proliferation of TNBC.
(a-c) T47D (a), BT-549 (b) and MDA-MB-231 (c) cells were treated as indicated and cell proliferation was measured. Error bars represent the ±SD of three independent experiments. Statistical analyses were performed with the ANOVA. #, $p<0.05$; ##, $p<0.01$. (d) Passage 3 tumors from HCI001 were used to test the effect of PD0332991 on metastasis. When primary tumors reached 100-150mm^3, mice were randomized and treated either with saline or PD0332991 for five weeks (n=8). Tumor volume was measured. Data are expressed as mean ± SD. Statistical analyses were performed with the Student's t-test. (e-f) MDA-MB-231 cells were injected into the mammary fat pad of immunodeficient mice. When tumors reached 400mm^3 in size, we removed the primary tumors and treated these mice with either vehicle or PD0332991 for an additional 12 weeks. Mice were sacrificed and number of metastatic lung nodules were counted and quantified. Data are represented as the means ±SD. Statistical analyses were performed with Student's t test. ##, $p<0.01$. (g) Purified recombinant GST, GST-CDK4, GST-CDK6 and His-SNAIL1 were incubated *in vitro* as indicated. The interaction between CDK4/6 and SNAIL1 was then examined. (h) Cells were transfected as indicated and treated with either vehicle or PD 0332991. Cell lysates were immunoprecipitated with HA antibody and western blotting was performed with indicated antibodies.

Supplementary Fig. 2

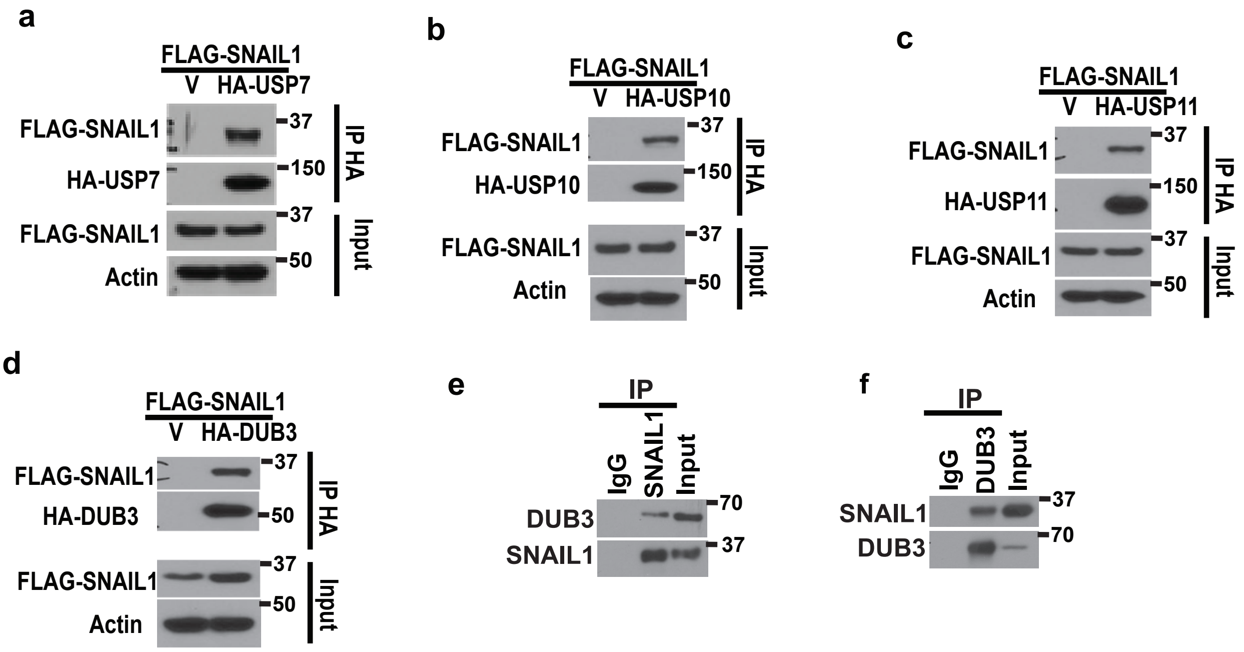

Supplementary Figure 2. DUBs interacts with SNAIL1.
HA-USP7 (a), HA-USP10 (b), HA-USP11 (c) and HA-DUB3 (d) were transfected in MDA-MB-231 cells stably expressing FLAG-SNAIL1. The co-IP experiment was performed using either an HA antibody to pull down HA-tagged deubiquitinases. Western blotting was performed with indicated antibodies. (e-f) BT-549 cell lysates were subjected to immunoprecipitation with control IgG, anti-SNAIL1 (e), or anti-DUB3 (f) antibodies.
The immunoprecipitates were then blotted with the indicated antibodies.

Supplementary Fig. 3

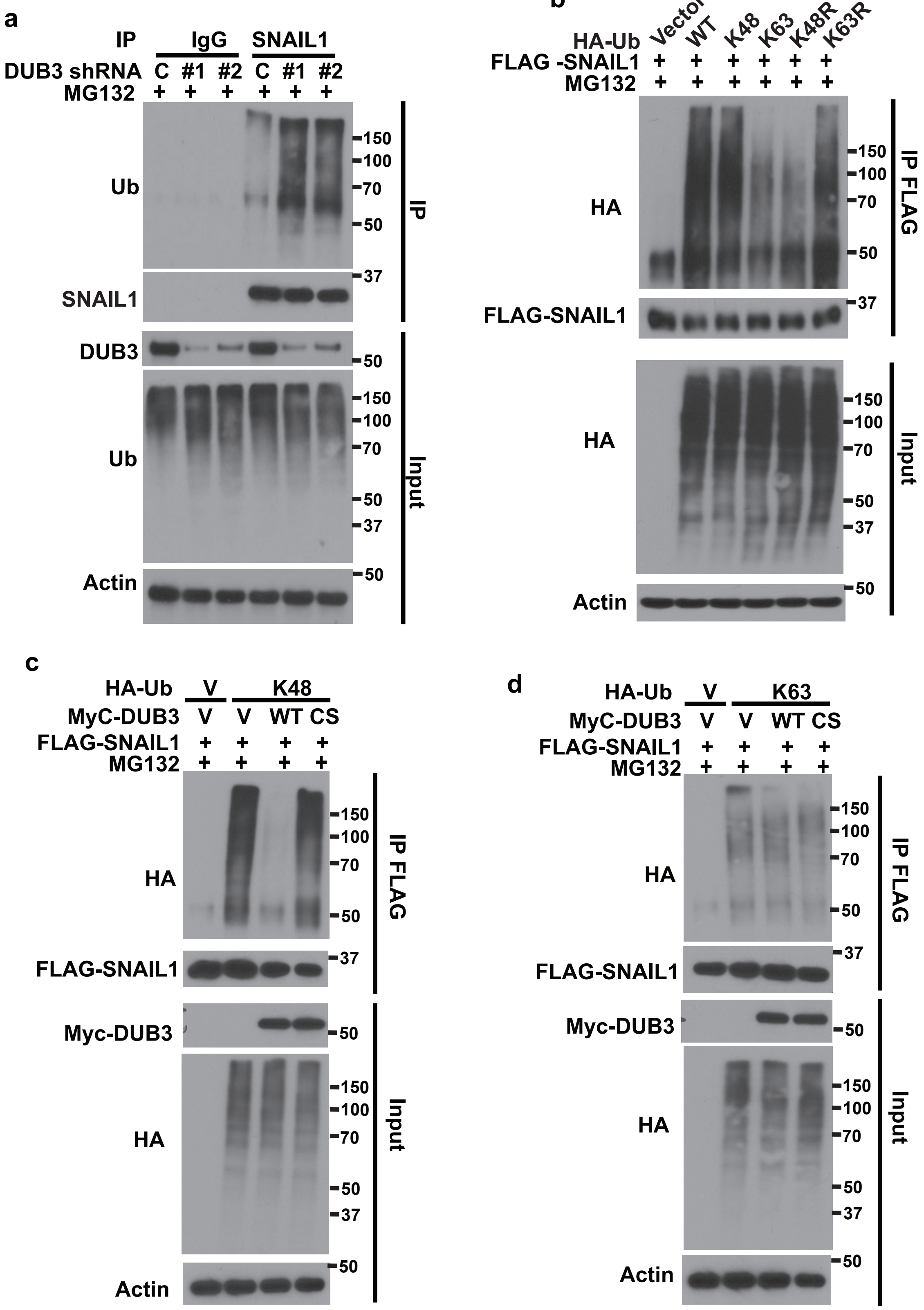

Supplementary Figure 3. DUB3 deubiquitinates SNAIL1

(a) MDA-MB-231 cells stably expressing control or DUB3 shRNAs were generated and cell lysates were subjected to immunoprecipitation with control IgG or anti-SNAIL1 and the polyubiquitylated SNAIL1 protein was examined.

(b) Type of ubiquitin linkage on SNAIL1. FLAG-SNAIL1 and indicated HA tagged ubiquitin were transfected. Cell lysates were boiled and immunoprecipitated with anti-HA beads and immunoblotted as indicated. (c-d) WT DUB3, not the CS mutant, decreases SNAIL1 K48 ubiquitination. Cells were transfected with indicated constructs. Cell lysates were boiled and immunoprecipitated with anti-FLAG beads and immunoblotted as indicated.

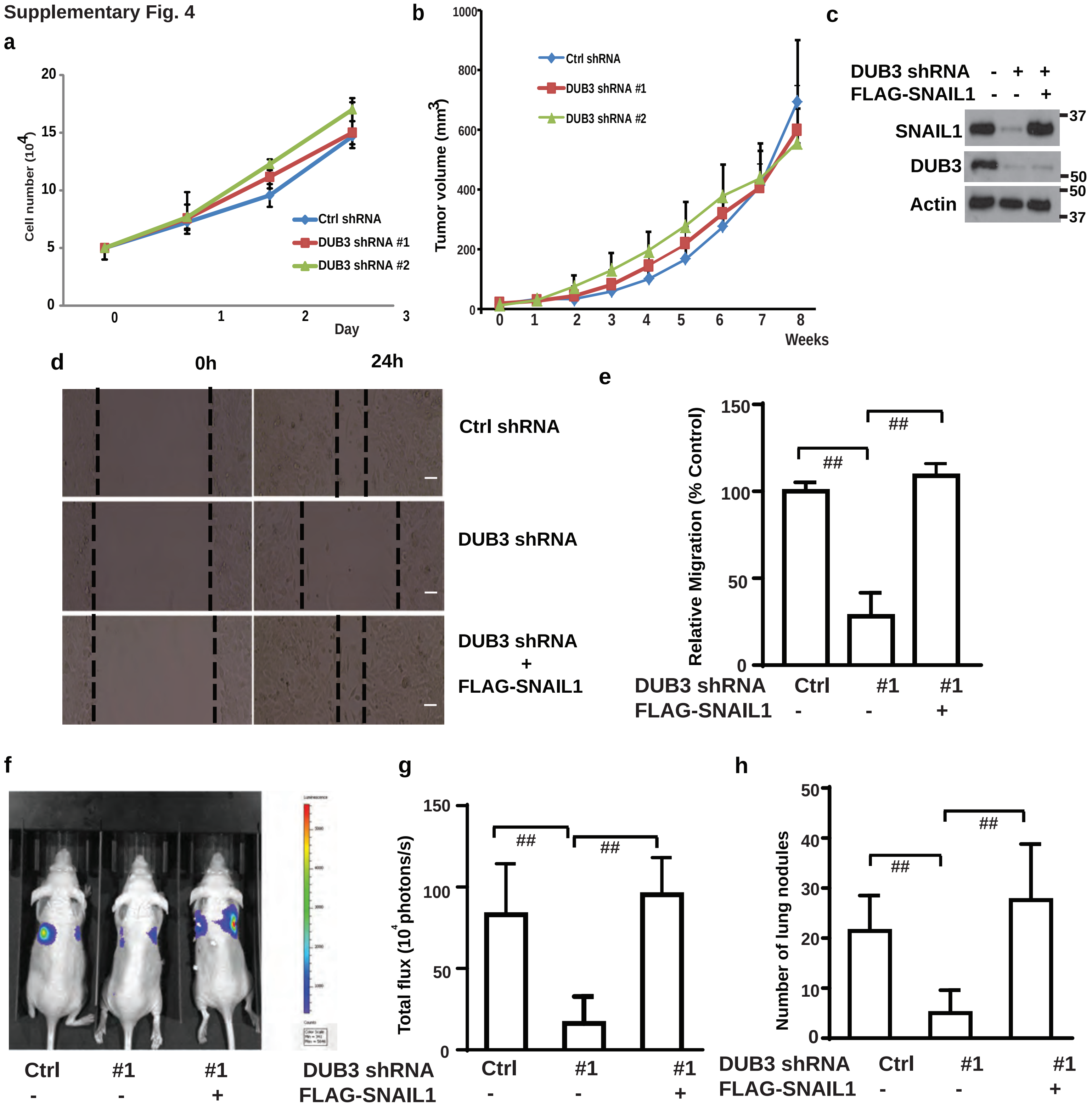

Supplementary Figure 4. DUB3 regulates the migratory activity of MDA-MB-231 cells via stabilizing SNAIL1. (a) MDA-MB-231 cells stably expressing control or DUB3 shRNAs were seeded in each well ($5x10^4$/well) and cell number was counted every 24 hour. Error bars represent the ± SD of three independent experiments. Statistical analyses were performed with the ANOVA. (b) Cells from (a) were injected into the mammary fat pad of immunodeficient mice. Tumor volume was measured. Data are expressed as mean ± SD. Statistical analyses were performed with ANOVA. (c) MDA-MB-231 cells stably expressing control or DUB3 shRNAs were transfected with indicated constructs. Western blot was performed with indicated antibodies. (d-e) The migratory ability of cells as in (c) was analyzed by wound healing assay and results were quantified in (e). The results represent the means ±SD of three independent experiments. Statistical analyses were performed with the ANOVA. ##p < 0.01. (f-h) One million cells from (c) were injected into the lateral tail vein of immuno-deficient mice (n=8). After 6 weeks, the development of lung metastases was recorded using bioluminescence imaging (f) and quantified (g).
After 12 weeks, mice were sacrificed and number of metastatic lung nodules was counted and quantified (h).
Data are represented as the means ±SD. Statistical analyses were performed with the ANOVA. ##, p<0.01.

Supplementary Fig. 5

a

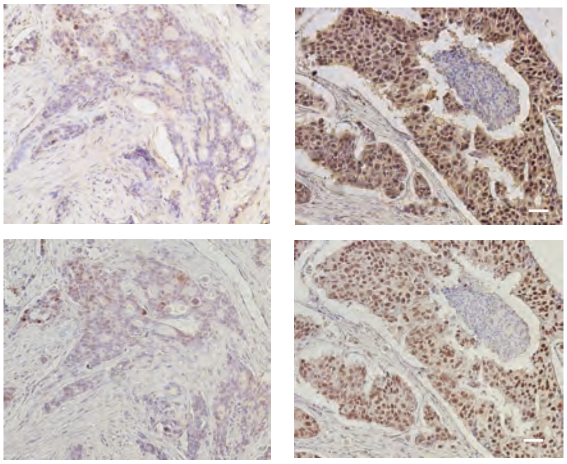

b

	DUB3-Low	DUB3-High	Total
Non-metastatic Carcinoma	48 (64%)	27 (36%)	75
Metastatic Carcinoma	30 (29%)	73 (71%)	103

P <0.0001 , R = 0.347

c

	SNAIL1-Low	SNAIL1-High	Total
Non-metastatic Carcinoma	41 (55%)	34 (45%)	75
Metastatic Carcinoma	22 (21%)	81 (79%)	103

P <0.0001 , R = 0.344

d

Metastatic Carcinoma:

	SNAIL1-Low	SNAIL1-High	Total
DUB3-low	19 (63%)	11 (37%)	30
DUB3-high	3 (4%)	70 (96%)	73

P<0.0001 , R = 0.657

e

	DUB3-Low	DUB3-High	Total
Basal like	10 (26%)	29 (74%)	39
Luminal	26 (67%)	13 (33%)	39

P <0.0001 , R = 0.411

f

	SNAIL1-Low	SNAIL1-High	Total
Basal like	7 (18%)	32 (82%)	39
Luminal	24 (62%)	15 (38%)	39

P <0.0001 , R = 0.445

Supplementary Figure 5 DUB3 expression positively correlates with SNAIL1 expression in Clinical Breast Cancer Samples

(a) Representative images of Immunohistochemical staining of DUB3 and SNAIL1 in non-metastatic and metastatic breast carcinoma. (b) Correlation study of the DUB3 expression level with metastatic carcinoma. (c) Correlation study of the SNAIL1 expression level with metastatic carcinoma. (d) Correlation study of DUB3 and SNAIL1 in metastatic carcinoma. Statistical analyses were performed with the χ2 test and Pearson correlation coefficient. (e) Correlation study of the DUB3 expression level with subsets of breast carcinomas. (f) Correlation study of the SNAIL1 expression level with subsets of breast carcinomas. Statistical analyses were performed with the χ2 test and Pearson correlation coefficient.

Supplementary Fig. 6

a

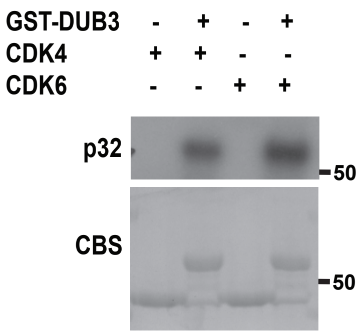

b

Sequence

TSLPEKS#PLSSEAR

Predicted Fragmentation Pattern

Seq	#	b: Δ Error	b	y	y: Δ Error	+1
T	1	---	102.055	---	---	14
S	2	---	189.087	1480.704	---	13
L	3	---	302.171	1393.672	---	12
P	4	---	399.224	1280.588	246.663	11
E	5	508.601	528.266	1183.535	-754.678	10
K	6	256.528	656.361	1054.493	227.009	9
S#	7	154.629	823.360	926.398	272.078	8
P	8	---	920.412	759.400	249.325	7
L	9	260.165	1033.497	662.347	169.744	6
S	10	102.051	1120.529	549.263	289.684	5
S	11	---	1207.561	462.231	480.202	4
E	12	141.864	1336.603	375.199	465.871	3
A	13	137.044	1407.640	246.156	487.426	2
R	14	---	---	175.119	---	1

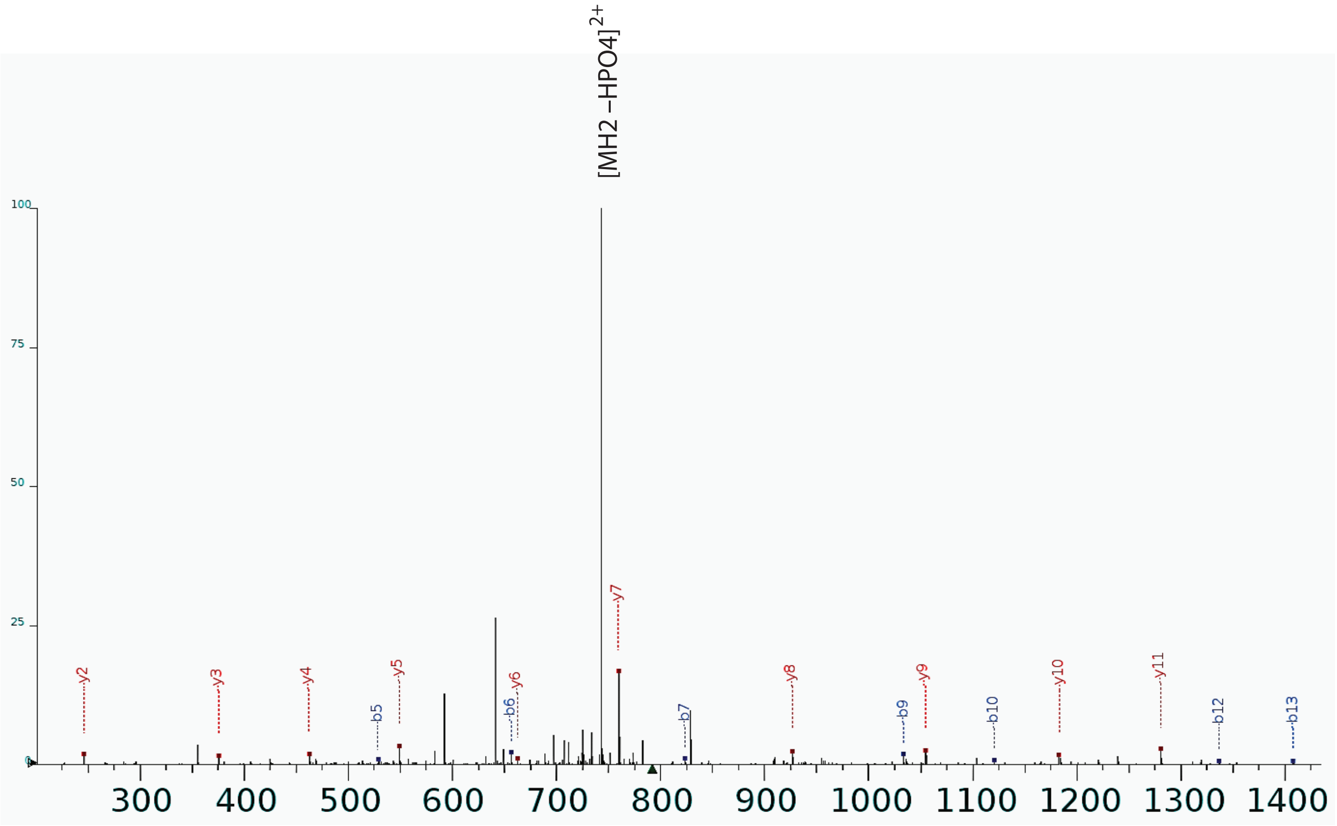

Supplementary Figure 6 (a) Bacterial expressed GST or GST-DUB3 WT fusion protein were incubated with active CDK4 or CDK6 in the presence of [γ-32P]ATP. Proteins were resolved by SDS–PAGE; phosphorylated proteins were visualized with autoradiography. (b) Identification of Ser41 phosphorylation in DUB3 by mass spectrometry analysis.

Supplementary Fig. 7

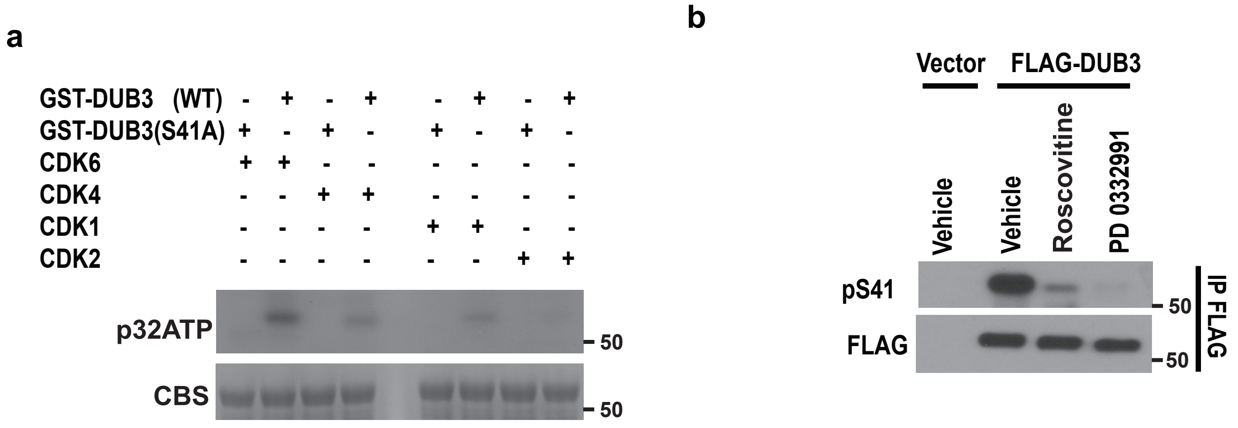

Supplementary Figure 7. CDKs phosphorylate DUB3 on Ser41

(a) CDKs phosphorylate DUB3 *in vitro*. Bacterial expressed GST-DUB3 WT and GST-DUB3 S41A fusion protein were incubated with active CDK4, CDK6, CDK1 or CDK2 in the presence of [γ-32P]ATP. Proteins were resolved by SDS–PAGE; phosphorylated proteins were visualized with autoradiography. CBS: Coomassie Blue Staining.

(b) Cells were transfected with indicated plasmids and were treated with vehicle, or pan-CDK inhibitor (Roscovitine) or CDK4/6 inhibitor (PD0332991). The phosphorylation of Ser41 was examined.

Supplementary Fig. 8

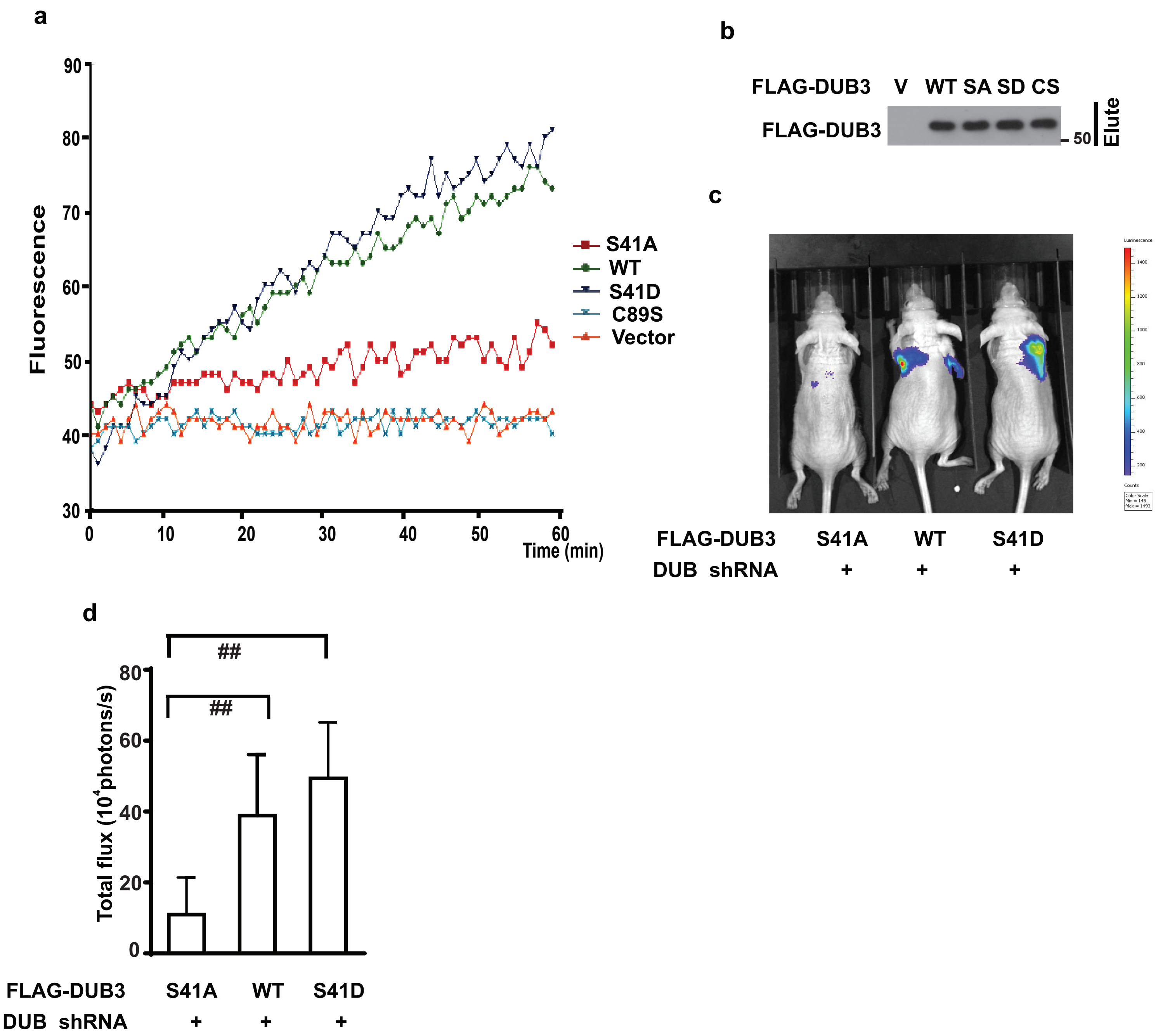

Supplementary Figure 8. Ser41 phosphorylation regulates DUB3 activity and cancer metastasis

(a) Cells transfected with indicated constructs were lysed and the co-IP experiment was performed using anti-FLAG antibody. The immunoprecipitates were eluted by FLAG-peptide containing buffer and the activity of DUB3 was measured using Ub-AMC as a substrate. (b) Elutes were detected with anti-FLAG antibody. (c-d) FLAG-DUB3 WT, S41A or S41D mutants were transfected in MDA-MB-231 cells stably expressing DUB3 shRNA. Cells were injected into the lateral tail vein of immuno-deficient mice (n=8). After 6 weeks, the development of lung metastases was recorded using bioluminescence imaging (c) and quantified (d). Data are represented as the means ±SD. Statistical analyses were performed with the ANOVA. ##, p<0.01.

Supplementary Fig. 9

Figure 1i

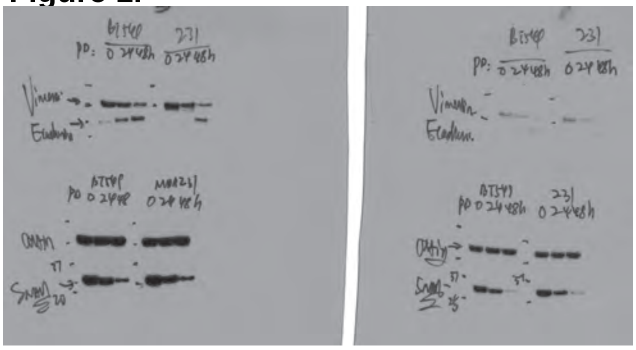

Figure 1j

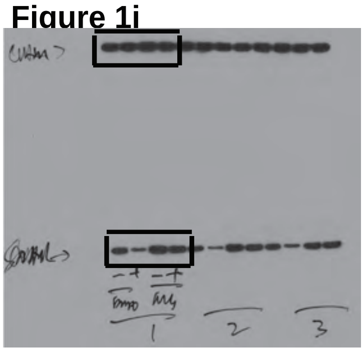

Figure 1l

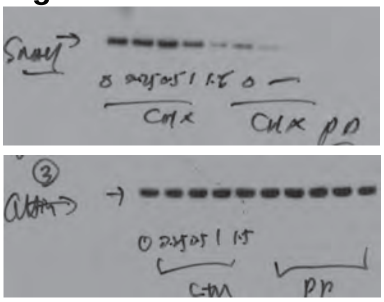

Figure 1n

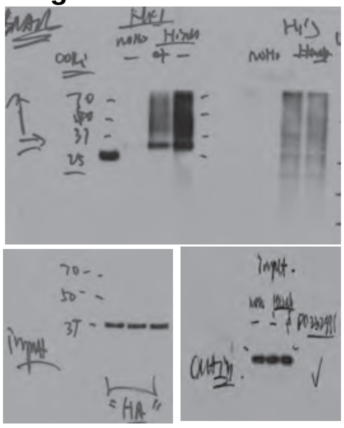

Figure 1o

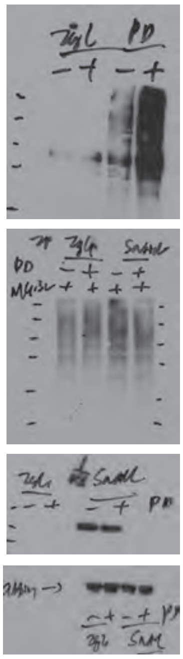

Figure 2b

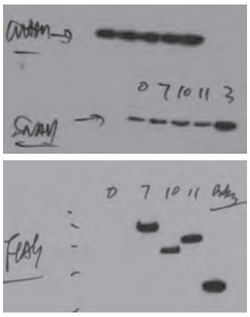

Figure 2c

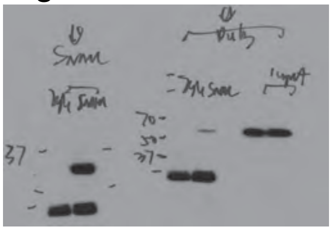

Figure 2d

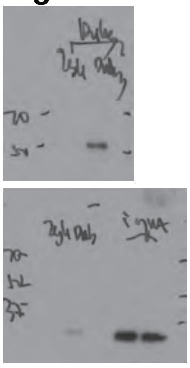

Figure 2e

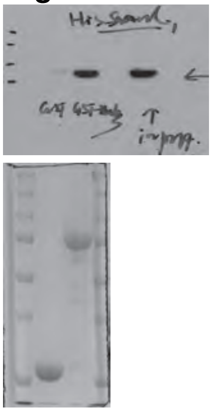

Supplementary Figure 9: Original scan of the blots presented a in the main text. Related to Fig1, and 2.

Supplementary Fig. 10

Figure 3a

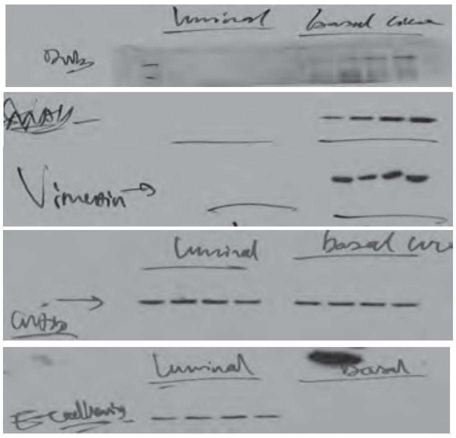

Figure 3b

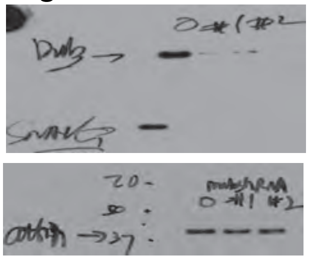

Figure 3d

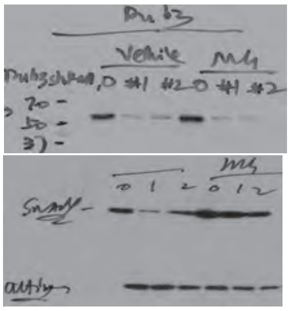

Figure 3e

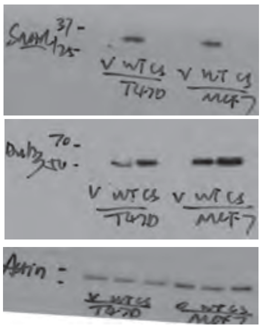

Figure 3f

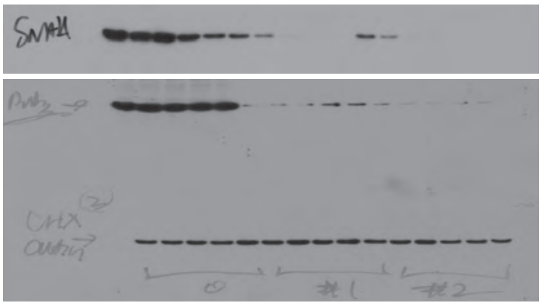

Figure 3j-i

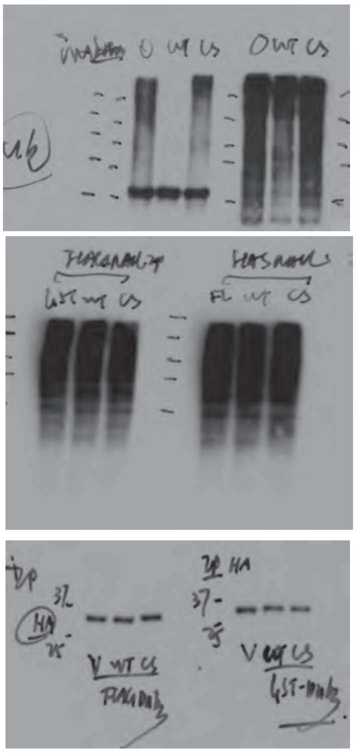

Figure 3h

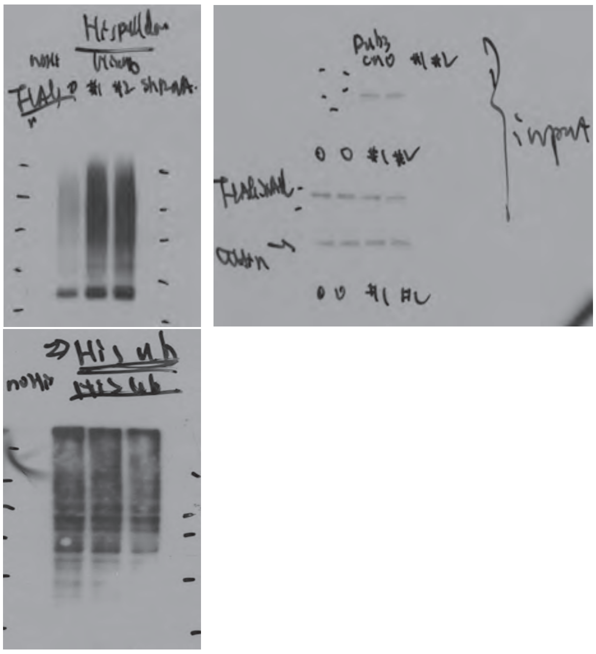

Supplementary Figure 10: Original scan of the blots presented a in the main text. Related to Fig 3.

Supplementary Fig. 11

Figure 4a

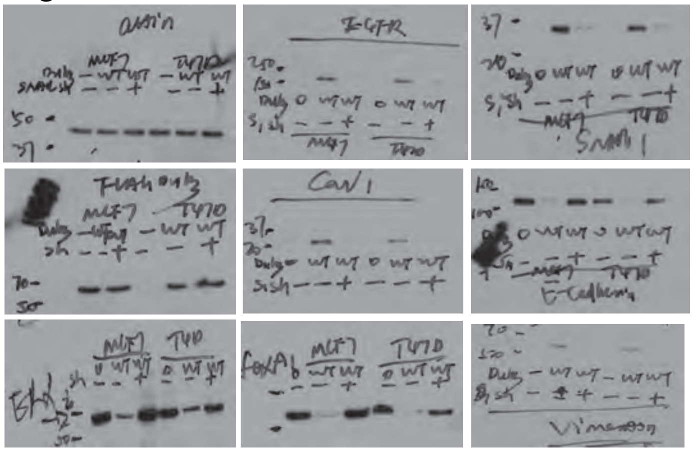

Figure 4c

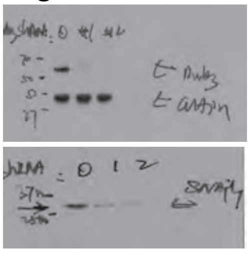

Figure 5a

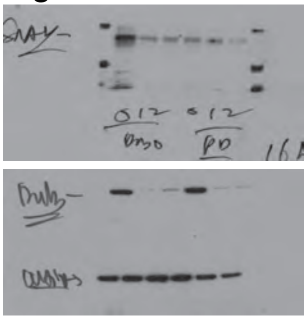

Figure 5b

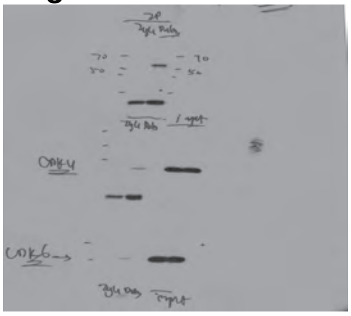

Figure 5c

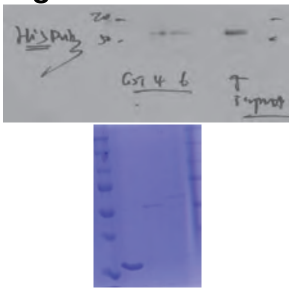

Figure 5d

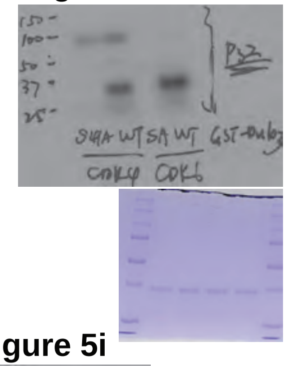

Figure 5e

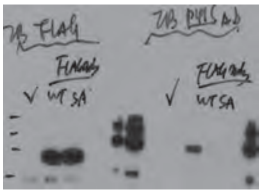

Figure 5f

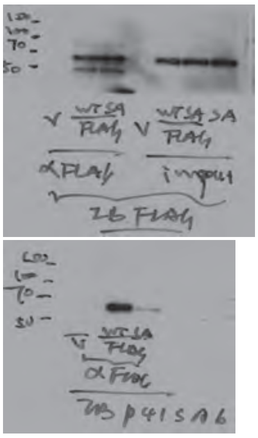

Figure 5g

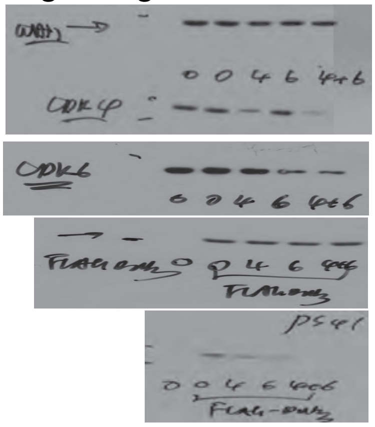

Figure 5h

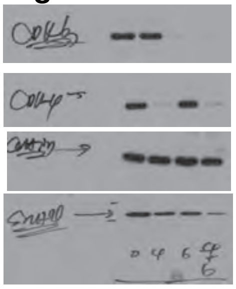

Figure 5i

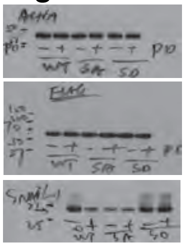

Figure 6a

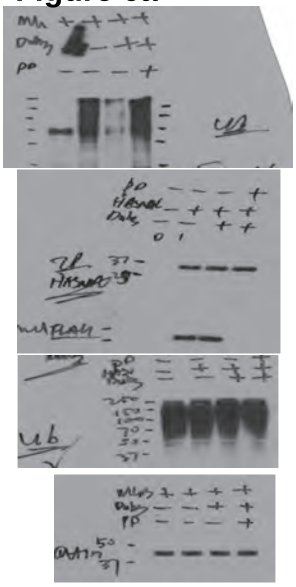

Figure 6b

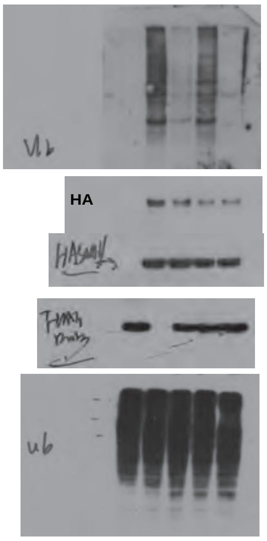

Figure 6c

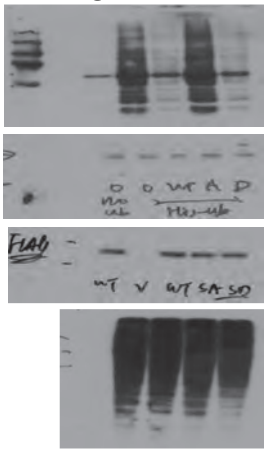

Figure 6d

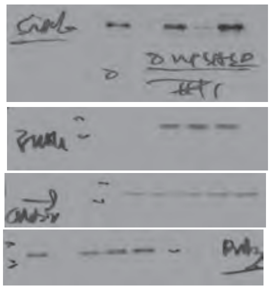

Supplementary Figure 11: Original scan of the blots presented a in the main text. Related to Fig 4, 5 and 6.

Supplementary Methods

In Vitro deubiquitinase Enzymatic Assay

In vitro enzymatic assays using ubiquitin-7-amido-4-methylcoumarin (Ub-AMC; U-550; Boston Biochem) as the substrate was performed in 50–100 µl reaction buffer (20 mM HEPES-KOH [pH 7.8], 20 mM NaCl, 0.1 mg/ml ovalbumin [A7641; Sigma], 0.5 mM EDTA, and 10 mM DTT) at 25°C. Fluorescence was monitored in an Infinite® M1000 PRO Fluorometer (TECAN). Ub-AMC is prepared by the C-terminal derivatization of ubiquitin with 7-amino-4-methylcoumarin and has been shown to be a useful and sensitive fluorogenic substrate for wide range of deubiquitinylating enzymes (DUBs), including ubiquitin C-terminal hydrolases (UCHs) and ubiquitin specific proteases (USPs).

Quantitative Real-Time PCR

Total mRNA was isolated from cells using PARIS Kit (Thermofisher). qPCR was performed using one-step, Brilliant SYBR Green qRT-PCR master mix kit (Stratagene) and Stratagene Mx3005P Real-Time PCR detection system (Stratagene) using primers against *Snail1*. All experiments were performed in triplicate with β-actin as an internal control. All samples were normalized to β-actin mRNA levels. Primer sequences are listed below. hSNAI1 F-ggccctggctgctacaaggc, R-ctcgagggtcagcggggaca; hACTB F-cgggaaatcgtgcgtgacatt R-tgatctccttctgcatcctgt.

Migration and Invasion assay

For migration assays (wound healing assays), cells were seeded in 6-well plates and grown until confluence. And then complete medium was replaced with serum-free medium for 24 h. Confluent cells (monolayer) was scraped with a P200 tip in each well, the medium was replaced with complete medium in the presence of Mitomycin C (10μg/mL) to block cell proliferation. After 24h, the cells were fixed with 3.7 % paraformaldehyde and photographs were obtained. For matrigel invasion assays, cells were seeded in 24-well invasion chamber (Corning, 354480). Each sample was plated in triplicate. All product recommended protocols were followed. To measure cell invasion, the filter was stained with 0.2 % crystal violet and invading cells were counted.

Animal studies

All animal work was done in accordance with a protocol approved by the Institutional Animal Care and Use Committee at Mayo Clinic. Female 5-7 week old SCID or Balb/c nude (NCI) mice were used. For PD0332991 experiment, MDA-MB-231 cells (1×10^6 cells/mouse) were injected into the mammary fat pad of mice. When tumors reached 400mm^3 in size, the primary tumors were removed and mice were divided into two groups by stratified randomization and treated with either vehicle or PD0332991 (150mg/kg daily, PO) for an additional 12 weeks. Mice were sacrificed and number of metastatic lung nodules was counted and quantified. For the lung metastasis study, MDA-MB-231 cells (1×10^6 cells/mouse) were transfected as indicated and injected into the lateral tail vein (8 mice/group). The development of lung metastases in mice with MDA-MB-231 was recorded using bioluminescence imaging at week 6. Mice with MDA-MB-231 cells were sacrificed at week 12 and visible lung metastatic nodules were examined macroscopically. For primary tumor growth experiment, MDA-MB-231 cells (1×10^6 cells/mouse) were injected into the mammary fat pad of mice and tumor volume was measured once per week. Data were analyzed using ANOVA or Student's t test. P value less than 0.05 was

considered significant. Mice were subjected to euthanasia if they displayed pain or distress, such as lethargy, lying down, not eating or drinking, weight loss greater than 10% body weight or difficulty breathing. According to the blinding procedures, two people as a group performed all the mice experiment. One person performed the experiments and another one, totally blinded to the experiment group, measured the tumor volume and weight, quantified number of lung and liver nodules, and analysed all data.

Tissue Microarray

The tissue arrays of breast cancer samples were purchased from US Biomax (BR1101, BR20838). Immunohistochemical staining of DUB3 (dilution 1:500) and SNAIL1 (dilution 1:500) were carried out using IHC Select® HRP/DAB kit (Cat. DAB50, Millipore). The immunostaining was scored by pathologists in a blinded manner. The IHC score was calculated by combining the quantity score (percentage of positive stained cells) with the staining intensity score. The quantity score ranges from 0 to 4, i.e. 0, no immunostaining; 1, 1－24% of cells are stained; 2, 25－49% are positive; 3, 50－74% are positive; and 4, $\geqslant$ 75% of cells are positive. The staining intensity was scored as: 0 (negative), 1 (weak), 2 (moderate) and 3 (strong). The score for each tissue was calculated by multiplying the intensity with the quantity score (the range of this calculation was therefore 0-12). An IHC score of 9－12 was considered a strong immunoreactivity; 5–8, moderate; 1–4, weak; and 0, negative. Samples with IHC score > 4 were considered to be high, and $\leqslant$4 were considered to be low. The χ2 test and the Pearson correlation coefficient were used for statistical analysis of the correlation between DUB3 and SNAIL1, and the correlation of DUB3 or SNAIL1 with cancer type.
